# Molecular Simulation Analysis of Polyurethane Molecular Structure under External Electric Field

**DOI:** 10.3390/molecules29184329

**Published:** 2024-09-12

**Authors:** Zhiyi Pang, Shangshi Huang, Yi Li, Yiyi Zhang, Rui Qin

**Affiliations:** 1 Faculty of Intelligent Manufacturing, Nanning University, Nanning 530200, China; pangzhiyi95@163.com; 2 State Key Laboratory of Power System, Department of Electrical Engineering, Tsinghua University, Beijing 100084, China; 3 School of Electrical Engineering, Guangxi University, Nanning 530004, China; 4 Liuzhou Power Supply Bur Guangxi Power Grid Co., Ltd., Liuzhou 545000, China

**Keywords:** polyurethane, molecular simulation, electric field application, molecular structure, power equipment

## Abstract

Polyurethane (PU) materials are extensively utilized in power equipment. This paper introduces a comprehensive evaluation method that combines electromagnetics and computational chemistry based on the Density Functional Theory (DFT) to elucidate the impact of external electric fields on the molecular structure of PU during electrical contact. The study focuses on the microstructural and molecular energy changes in the hard (HS) and soft (SS) segments of PU under the influence of an electric field of uniform intensity. Findings indicate that the total energy of HS molecules decreases markedly as the electric field intensity increases, accompanied by a significant rise in both the dipole moment and polarizability. Conversely, the total energy and polarizability of the SS molecules decrease, while the dipole moment experiences a slight increase. Under the influence of a strong electric field, HS molecules tend to stretch towards the extremities of the main chain, leading to structural instability and the cleavage of hydroxyl O-H bonds. Meanwhile, the carbon chain of the SS molecules twists towards the center under the electric field, with no chemical bond rupture observed. At an electric field intensity of 8.227 V/nm, the HOMO-LUMO gap of the HS molecule narrows sharply, signifying a rapid decline in the molecular structure stability, corroborated by infrared spectroscopy analysis. These findings offer theoretical insights and guidance for the modification of PU materials in power equipment applications.

## 1. Introduction

Polyurethane (PU) is a polymer material synthesized through the addition polymerization of monomers like polyisocyanates, polyols, and polyamines [1]. The distinctive microphase separation between the incompatible polyol microregions (soft chain segments, SSs) and carbamate microregions (hard chain segments, HSs) endows PU with a unique structure [2]. This structure imparts to PU an array of exceptional physical and mechanical properties, such as high elasticity, superior low-temperature flexibility, excellent wear resistance, and robust adhesion [3]. The SS molecule, primarily composed of polyols, is pivotal in enhancing the tensile strength, elongation at break, and tear resistance of PU [4]. The HS molecule, derived from isocyanates and chain extenders, contributes to the material’s comprehensive properties, including strength and toughness, through its microphase separation structure [5]. By meticulously adjusting the proportions of HSs and SSs in PU [6], a variety of products with tailored physical and mechanical properties can be fabricated [7,8].

PU materials are extensively employed in the protective and supportive structures [9] of power equipment due to their exceptional insulating, mechanical, and chemical properties [10,11]. In the practical application of these materials within power equipment [12,13,14], electrical contact is often unavoidable. Such contact can induce changes in the electric field distribution, potentially affecting the intrinsic characteristics and properties of the PU. Distortions in the electric field may concentrate charges at specific points, potentially causing partial discharges. This could further initiate an electron avalanche effect within the polymer coating, leading to the formation of dendritic microchannels and ultimately causing device damage. However, the molecular structural degradation mechanisms of polyurethane under the influence of external electric fields are not fully understood. Further research is essential to clarify these mechanisms.

In the realm of high-voltage insulation technology, insulating materials are typically assessed through experimental methods. However, due to operational conditions and impurities, the degradation characteristics of insulating materials in actual service cannot be precisely characterized. Thus, molecular simulation methods are essential, offering a predictive capability at the molecular and atomic levels. These methods can efficiently and cost-effectively guide the design of new materials and the optimization of existing ones [15,16]. This paper employs molecular simulation to investigate strategies [17] for enhancing the resistance of power equipment to intense electric fields and to forecast the vulnerabilities of HS and SS molecules under such conditions. Concurrently, the molecular structures of HS and SS molecules were calculated and analyzed using a semi-empirical approach in conjunction with Gaussian 09W [18] and Multiwfn software [19,20]. The impact of electric field polarization on these molecules was examined through parameters such as the polarizability, dipole moment, frontier orbitals, and infrared (IR) spectra under a uniform external electric field. Utilizing these data [21], the weak points within each molecule were identified. These studies offer a theoretical underpinning for the enhancement of PU coatings in the protection of power equipment, serve as a guide for micro-level modifications of PU coatings [22,23], and contribute to the advancement in insulating coating technologies for power equipment.

## 2. Method

### 2.1. Model Construction

**Figure 1:**
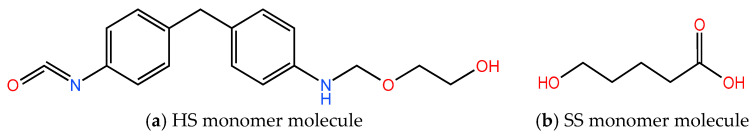
HS (**a**) and SS (**b**) molecular structure formula of PU.

The core properties of PU are predominantly determined by its hard chain segments, which significantly influence the physical, mechanical, and optical characteristics of the polymer [24,25]. In the chemical industry [26], HSs are commonly synthesized from aromatic isocyanates [27]. Studies have shown that toluene diisocyanate (TDI) and methylene diphenyl diisocyanate (MDI) [28] are the two most prevalent aromatic isocyanates used. MDI [29], in particular, is favored for its superior physical and chemical properties [29,30] due to the high symmetry of its molecular structure [27]. In this molecular simulation experiment [31], the HS molecule is created by reacting MDI with ethylene glycol [32], and the resulting monomer molecular structure is depicted in Figure 1a.

Conversely, the SSs of PU are primarily composed of polyester or polyether chains with relatively low molecular weights, which are crucial in determining the elasticity and flexibility of PU materials [33,34]. In this study, poly(hydroxyalkanoate) (PHA) is chosen as the SS molecule, and its monomer molecular structure is illustrated in Figure 1b. This approach allows us to investigate the impact of varying chain segment structures on the overall properties of PU.

### 2.2. Theoretical Calculations and Methods

**Figure 2:**
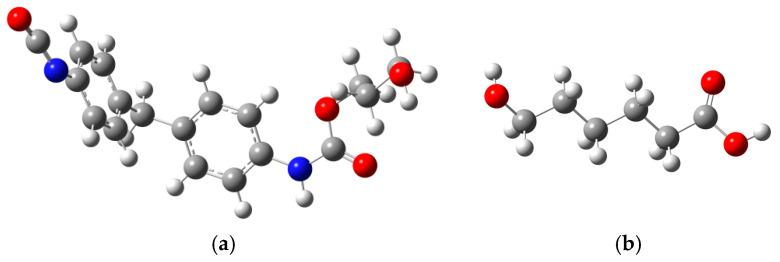
(**a**) HS molecular optimization model of PU; (**b**) SS molecular model optimization model of PU (Color coding: White—Atom H, Gray—Atom C, Blue—Atom N, Red—Atom O).

The objective of this study is to construct molecular models for the SS and HS of PU using molecular simulations, which is based on quantum chemistry. The goal is to track the evolution of the electronic structure and the alterations in the molecular core framework. The methodology of the research is outlined in the following steps:

As shown in Figure 2a,b, the molecular geometry of the PU’s SS and HS models is optimized using the B3LYP functional set with a 6-31G basis within the framework of density functional theory (DFT) [35]. In this paper, a visualization of the molecular ball and stick model is realized by GaussView 5.0. This process aims to achieve a stable molecular configuration at the lowest energy state. The compounds in question are characterized by neutral and singlet charge and spin states, necessitating the use of the unrestricted Kohn–Sham method for the calculations. The DFT optimization results indicate that the SS and HS molecules of PU have attained the lowest total energy, with no imaginary frequencies detected. This absence of imaginary frequencies confirms that the Maximum Force, RMS Force, Maximum Displacement, and RMS Displacement in the computational outcomes have all reached convergence.

Furthermore, in our simulation experiments, we employed the semi-empirical PM6 method [36] and introduced an electric dipole field along the *X*-axis of the molecular chain, ranging from 0 to 0.0160 atomic units (a.u., where 1 a.u. is equivalent to 5.142 × 10^11^ V/m). This was performed to refine the model and compute the single-point energy, with the objective of uncovering the microscopic mechanisms behind the progressive degradation of the SS and HS molecular constituents of PU under the influence of an external electric field.

All simulations and calculations were executed using the Gaussian 09W software suite, and a comprehensive data analysis was conducted with the Multiwfn 3.7 software. These findings offer valuable theoretical insights into the behavior of PU materials when subjected to external electric fields.

## 3. Simulation Results and Discussion

### 3.1. Effect of External Electric Field on Molecular Dipole Moment, Energy, and Polarizability

**Figure 3:**
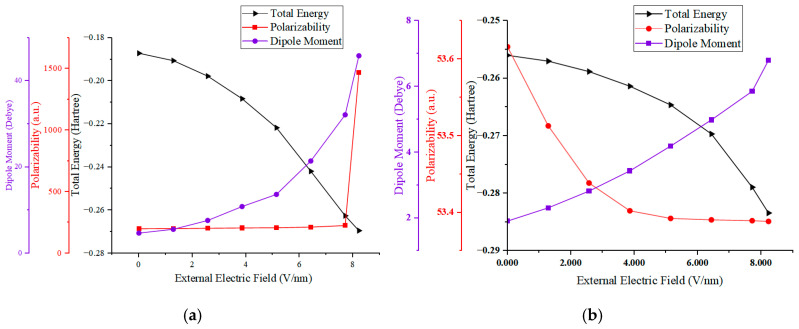
The external electric field–dipole moment, polarizability, and total energy of HS (**a**) and SS (**b**) molecules of PU.

A meticulous examination of parts (a) and (b) in Figure 3 reveals clear trends in the variations in the dipole moment and total energy for both the SS and HS molecules. As the electric field intensity increases, the total energy of these molecules diminishes continuously, with the extent of this reduction expanding in tandem with the field’s intensity. This behavior can be ascribed to the progressive migration of electrons in the molecular outer shell under the influence of the external electric field.

Polarizability, as depicted in Figure 3, quantifies the degree to which a dielectric material becomes polarized in response to an external electric field. It is a critical parameter that correlates directly with the insulating capabilities of the material; a higher polarizability equates to inferior insulating properties. Microscopically, this suggests that the bound charges within the dielectric are becoming liberated from atomic constraints and are transitioning into a free state. Macroscopically, an increase in polarizability signals a degradation in the material’s dielectric performance.

Specifically, as illustrated in Figure 3a, both the dipole moment and polarizability of the HS molecule exhibit a sharp upward trend. The dipole moment soars from 4.2 to 50.7, and the polarizability jumps from 199.7 to 1467.5. Notably, these values experience a significant shift at an external electric field strength of approximately 7.7 V/nm, suggesting that the HS molecule’s breakdown threshold lies between 7.7 and 8.227 V/nm (equivalent to atomic units ranging from 0.0155 to 0.016).

Conversely, in Figure 3b, the SS molecule’s dipole moment rises from 1.95 to approximately 6.8, while its polarizability slightly decreases from 53.6 to around 53.4. At an electric field strength of 8.227 V/nm (0.016 a.u.), no breakdown of the SS molecule was detected, indicating that this field strength does not represent the SS molecule’s breakdown threshold.

Thus, it can be seen that the conclusion of Equations (1) and (2) is as follows:

(1) $$H=H_{0}+H_{int}$$

(2) $$H_{int}=-\mu \times F$$

In this study, we conduct an in-depth quantitative analysis to explore the intricate relationship between a molecule’s total energy, free energy, and interaction energy, as delineated by Equation (1). Equation (2) further clarifies the inverse correlation between a molecule’s interaction energy and the strength of the electric field. By integrating these equations, we deduce that an increase in the external electric field strength directly results in a reduction in total molecular energy, with the extent of this reduction being influenced by the molecular dipole moment.

For the SS and HS molecules of PU, their distinct dipole moments lead to a pronounced divergence in their polarizability trends under the same electric field strength. Specifically, the HS molecules, which possess larger dipole moments, are more prone to polarization under the influence of an electric field, thereby exhibiting higher polarizability. Conversely, the SS molecules may experience geometric distortions that lead to a decrease in polarizability [37].

Quantitative analysis reveals that the polarization of SS and HS molecules in response to an applied electric field is intimately connected to their dipole moments. This correlation not only sheds light on how molecular structure impacts the electrical properties of materials but also lays a preliminary theoretical foundation for the development of PU materials with enhanced insulation capabilities.

### 3.2. Effect of External Electric Field on the Geometry of the Molecule

**Figure 4:**
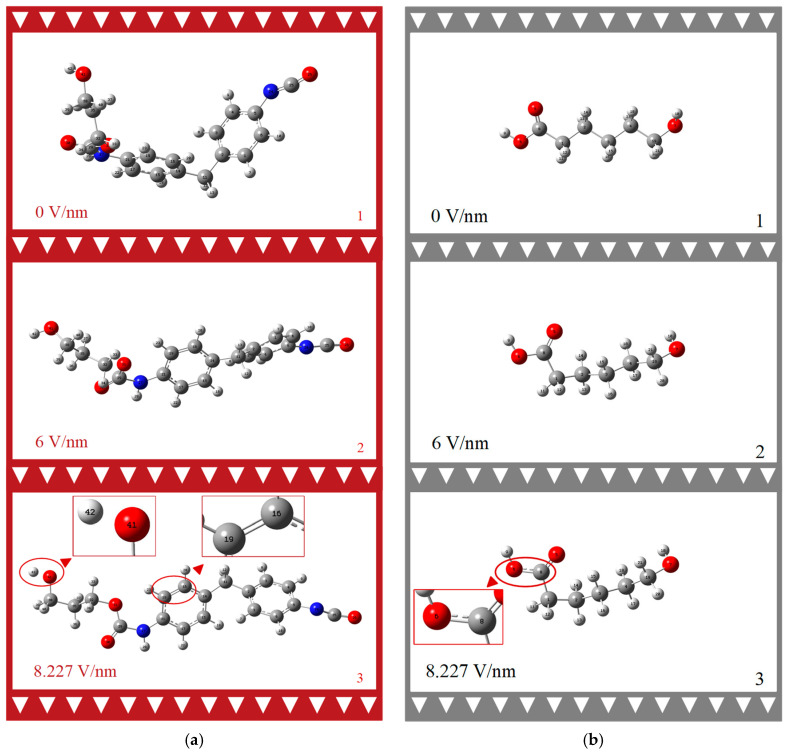
Geometric structure change process of PU HS (**a**) and SS (**b**) molecules under external electric field. (Color coding: White—Atom H, Gray—Atom C, Blue—Atom N, Red—Atom O. Red triangles are indication marks for local amplification).

Upon close examination of Figure 4a,b, it is evident that both the HS and SS molecules undergo significant geometric changes under the influence of an external electric field. Figure 4a illustrates that the HS molecule expands from a twisted state to a linear configuration along the *X*-axis under the external electric field, eventually adopting a net-like structure. Specifically, in the third image of series (a), at an external electric field of 8.227 V/nm, the hydroxyl groups H42 and O41 in the HS molecule dissociate, and the bond length between the C16 and C19 atoms on the benzene ring shifts from a single bond to a double bond. In contrast, the SS molecule’s neat, straight chain structure is distorted into an irregular form, with the C-O bond length between O6 and C8 increasing to 1.5 Å. Even under an 8.227 V/nm external electric field, the SS molecules do not exhibit geometric collapse or chemical bond rupture.

The dihedral angle is employed to measure the degree of molecular twisting under the external electric field, as detailed in Table 1. The table’s second column presents the dihedral angles formed by the carbon atoms C14 and C2 on the two benzene rings connected to the methyl group, and C21 and C5 on the diagonal. The third column pertains to the carbon chain C32, C35, and C38 of the chain extender and the terminus O31. Dihedral angle D1 increases from −2.43° to 8.19°, then decreases to −22.03°. D2 rises from −172.83° to −42.24° and subsequently decreases to −177.97°. These data reflect the structural response of the MDI-based PU hard chain molecules to an 8.227 V/nm electric field. The dihedral angle data alone suggest that the molecular structure maintains excellent macroscopic physical properties, such as high toughness and wear resistance. Regarding D3, the carbon atoms C8, C1, C2, and C3 on this linear chain do not break down; instead, they only undergo a deformation of 110.40°.

Based on this analysis, we can infer that, under the influence of a strong electric field, the HS and SS molecules, which exhibit opposing external appearances, may lead to irreversible macroscopic deformations, such as the cracking or flaking of PU coatings. If the HS content is relatively high, it could result in curing layer rupture, significantly reducing insulation and corrosion resistance.

### 3.3. Effect of External Electric Field on Molecular Front Orbitals Space Charge Properties of HS and SS of PU

The HS and SS of PU possess distinct properties compared to other dielectric materials. As per the analysis, the elastic HS tends to align with the main chain direction under the influence of a strong electric field, while the pliable SS bends towards the center under identical conditions. This molecular chain deformation is presumed to be the primary cause of PU material degradation. In the context of the frontier molecular orbital theory [38], the highest occupied molecular orbital (HOMO) and the lowest unoccupied molecular orbital (LUMO) characterize the electronic properties of molecules at their peak and trough energy states [39], respectively. The energy gap (EG) separating HOMO and LUMO serves as a metric for a molecule’s electron transfer capability. An attenuated EG implies a heightened propensity for intermolecular chemical reactions and electron transition from LUMO to HOMO.

**Figure 5:**
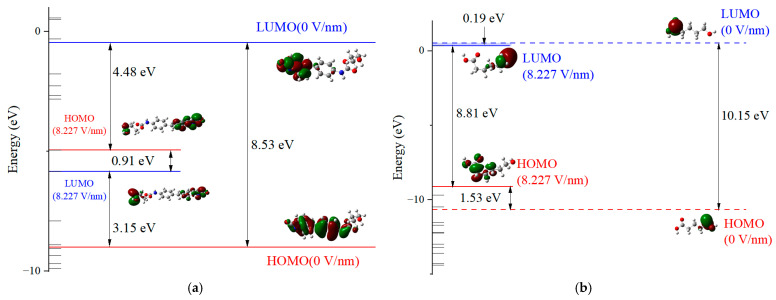
HS (**a**) and SS (**b**) molecule energy level and orbital distribution of PU under external electric field.

Figure 5a,b demonstrate that at an electric field of 0, the EG values for the PU’s HS and SS molecules are 8.53 eV and 10.15 eV, respectively. Upon elevating the applied electric field to 8.227 V/nm, these EG values diminish to 0.91 eV and 8.81 eV, respectively. The influence of an external electric field on molecular energy gaps can be reflected by the shift of molecular frontier orbitals. Before and after the application of an external electric field, the visualization results clearly demonstrate the trend of frontier orbital transfer and the degree of overlap with the HOMO/LUMO orbitals. The frontier orbitals of the HS molecule undergo a significant shift, leading to a proliferation of the asymmetric anti-bonding (π) orbitals at the molecular termini and a very high orbital overlap rate. This implies that molecular bonds are more susceptible to breaking and will be accompanied by structural changes. In contrast, the symmetric bonding (σ) orbitals at the ends of the SS molecule indicate a minimal orbital overlap rate, suggesting that electron transfer is less likely and structural changes are more probable [40]. This reduction in EG suggests an increased feasibility of electron movement along the molecular chain, augmenting the potential for chemical reactions to transpire along the chain and precipitating alterations or even disruptions in chemical bonds. Prolonged exposure to a potent electric field can incite a chain reaction, culminating in a degradation of the dielectric material’s electrical and mechanical properties and potentially initiating an avalanche breakdown.

**Figure 6:**
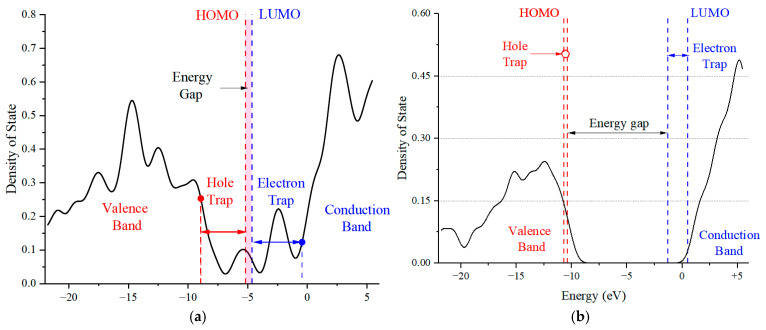
Energy level distribution and state density diagram of HS (**a**) and SS (**b**) molecules in PU under external electric field.

Figure 6a,b present the density of states (DOSs) diagrams generated by Gaussian 09W and analyzed with Multiwfn 3.7 software. These visual representations offer insights into the microscopic traits of the HS and SS molecules of PU, highlighting the trap energy levels as seen from an alternative perspective. Subject to an electric field, both the HS and SS molecular chains manifest a variety of deep and shallow energy levels in proximity to the valence and conduction bands. As the electric field intensity augments, the HOMO energy level rises, introducing additional hole traps within the valence band, while the LUMO energy level descends, creating more electron traps in the conduction band. The prevalence of electron traps over hole traps in both the HS and SS molecules suggests a greater propensity to capture free electrons under the influence of an external electric field, which can lead to a distortion in the spatial charge distribution within the dielectric material. Nonetheless, the SS molecule exhibits a significantly higher EG than the HS molecule, signifying greater stability under strong electric fields. This observation implies that in the design of PU for power equipment, the proportion of SS could be increased, provided that the mechanical properties of the material are preserved.

Table 2 delineates the top three atoms contributing most significantly to the premolecular orbital occupancy at an electric field strength of 8.227 V/nm. These data substantiate the validity of the results from an alternative perspective. In the case of the HS molecule’s HOMO, the terminal hydroxyl group’s O41 atom exhibits a substantial contribution of 54.52%, while the combined contribution of C38 and C25 amounts to 20.03%. Within the LUMO, the atoms C25 and O41, which coincide with those in the HOMO, facilitate electron transition from HOMO to LUMO. This implies that the molecular geometry is susceptible to instability under a persistent electric field, with the chemical bond at O41 being most prone to rupture. In the SS molecule, although the HOMO and LUMO do not share overlapping atoms, the O5 and O7 atoms are particularly susceptible to potential bond alterations.

### 3.4. Molecular IR Spectra under Different Electric Field Intensities

**Figure 7:**
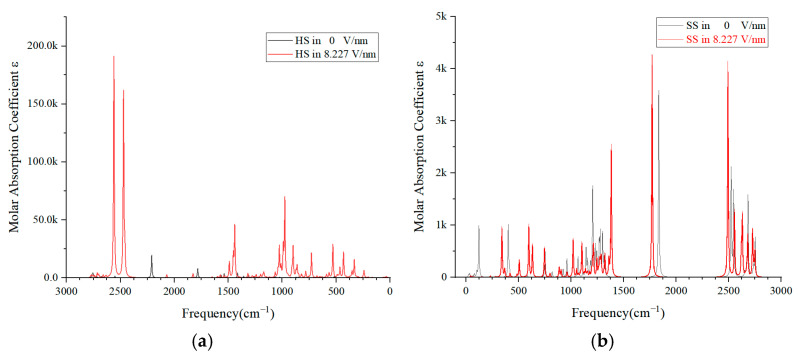
Molecular infrared spectra of HS (**a**) and SS (**b**) of PU under external electric field.

In this study, we utilized Multiwfn 3.7 software to further process the outcomes of molecular simulations, conducting an analysis of the IR spectra for the HS and SS of PU. This analysis was performed using the default parameters and a frequency correction factor of 0.96. As depicted in Figure 7a, at an applied electric field intensity of 8.227 V/nm, there is a notable increase in the fluctuation of the IR spectra compared to the scenario with no electric field. This increase suggests a significant structural impact on the HS molecule. Typically, the IR spectrum, as presented in Figure 7, concentrates on the mid-infrared region, with absorption peaks ranging from 4000 to 400 cm^−1^, indicative of molecular vibrations and accompanying rotational phenomena.

The prominent double peaks with amplitudes reaching 180 k observed at 2500 cm^−1^ are primarily due to the O-H bond in the associated state, and their presence clearly indicates the disruption of the hydroxyl groups H42 and O41 within the HS molecule. The absorption peak at 1500 cm^−1^ is typically linked to the vibrations of the C-H and N=O bonds in the aromatic molecule, signifying the alterations in bond energy on the benzene ring. The absorption peaks ranging from 1000 to 500 cm^−1^ are predominantly associated with the stretching or bending of C-O bonds and polar bonds. Additionally, the peak at 400 cm^−1^ is largely related to molecular rotation. The comparative analysis of the IR spectrum before and after the application of the external electric field indicates that the HS molecule is in a highly unstable state under the influence of an 8.227 V/nm electric field.

Figure 7b illustrates that the peak amplitude of the SS molecules typically remains below 5 k. Even with the presence of both broad and sharp peaks within a certain spectral range, the displacement of these peaks is minimal with or without the application of an electric field. This observation suggests that SS molecules exhibit excellent vibrational absorption capabilities and maintain a relatively stable molecular configuration when subjected to an electric field.

A comparative analysis of the IR spectra peaks and their ranges between Figure 7a,b allows us to deduce that an increasing electric field intensity could potentially lead to structural failure in the HS molecule [41]. Under the persistent influence of a strong electric field, the HS molecules might become the primary site of degradation for the PU material. The progressive degradation of HS molecules could generate a multitude of free radicals, which, under continuous electric field influence, might culminate in the catastrophic failure of the polymer’s insulation properties.

The findings of this study demonstrate a strong alignment between the IR spectral analysis and the conclusions drawn from the preceding sections of this chapter. This concordance underscores the potential of leveraging molecular simulation analysis to compare with real-world scenarios, thereby making significant contributions to the advancement of non-destructive testing methods for PU coatings [42].

## 4. Conclusions

The molecular simulation studies detailed in this manuscript provide a deep understanding of how PU materials behave under the influence of external electric fields. This aligns with our initial goal to delve into the molecular basis of PU’s insulation properties. Our findings yield the following key conclusions that contribute to the understanding of PU’s interaction with electric fields:

(1) The pronounced rise in polarizability and dipole moment of the HS molecules at specific electric field intensities indicates their intrinsic instability, which in turn affects the overall stability of PU materials. This insight is fundamental to grasping the structural dynamics within PU chains and underscores the importance of material optimization for improved stability.
(2) Our examination of molecular bonds and dihedral angles has revealed the distinct mechanical and electrical stabilities between SSs and HSs. The greater electrical stability of SS molecules, even amidst their mechanical vulnerability, suggests opportunities for material modification. This could particularly enhance PU’s electrical properties through targeted alterations to terminal hydroxyl or hydrogen atoms.
(3) The notable reduction in the energy gap of the HS molecules with increasing electric field intensity signals their increased risk of dielectric breakdown. The finding that a higher proportion of SS molecules can mitigate this effect offers a strategic pathway for developing PU materials with superior insulation properties, which could enhance the reliability of power equipment.
(4) The IR spectral analysis confirms the pivotal role of HS molecules in the degradation of PU materials under electric fields. The spectral variations provide a predictive tool for non-destructive testing, which could significantly decrease labor costs and increase the efficiency of engineering practices.

In conclusion, our research has elucidated the complex interplay between the molecular structure and the electrical properties in PU materials and has set the stage for the creation of novel insulation materials with customized properties. The conclusions from our study are anticipated to inspire further research and innovation in polymer science and its electrical engineering applications.

## Figures and Tables

**Table 1:** Dihedral angle of HS and SS of PU under external electric field.

Electric Field Intensity [V/nm]	HS		SS
	D1 (C21, C14, C2, C5) [°]	D2 (O31, C32, C35, C38) [°]	D3 (C8, C1, C2, C3) [°]
0	−2.43	−172.83	175.03
1.2855	−3.42	−164.25	172.38
2.571	3.31	−58.68	168.46
3.8565	4.18	−58.55	163.34
5.142	7.00	−61.31	154.79
6.4275	8.19	−59.53	140.53
7.713	−7.93	−42.24	114.99
8.2272 (0.016 a.u.)	−22.03	−177.97	64.63

**Table 2:** Contribution rate of orbital occupation of PU molecule.

HH Molecule				SS Molecule			
HOMO		LUMO		HOMO		LUMO	
O41	54.52%	C25	29.31%	O5	63.64%	O7	42.23%
C38	13.19%	O41	15.67%	O6	13.73%	C19	39.04%
C25	6.84%	O26	14.75%	C1	8.50%	H10	6.27%

## Data Availability

The data that support the findings of this study are available from the corresponding author upon reasonable request.
